# Cooling during transportation of newborns with hypoxic ischemic encephalopathy using phase change material mattresses in low-resource settings: a randomized controlled trial in Hanoi, Vietnam

**DOI:** 10.1186/s12887-024-04987-6

**Published:** 2024-08-08

**Authors:** Hang. T. T. Tran, Dien. M. Tran, Ha. T. Le, Lena Hellström-Westas, Tobias Alfvén, Linus Olson

**Affiliations:** 1 Department of Global Public Health, Karolinska Institutet, Stockholm, Sweden; 2 Vietnam National Children’s Hospital, Hanoi, Vietnam; 3 Department of Women’s and Children’s Health, Uppsala University, Uppsala, Sweden; 4 Sachs’ Children and Youth Hospital, Stockholm, Sweden; 5 Department of Women’s and Children’s Health, Karolinska Institutet, Stockholm, Sweden

**Keywords:** Asphyxia, Cooling, Encephalopathy, Low-income setting, Phase change materials

## Abstract

**Objective:**

To determine the effectiveness of phase-change-material mattress (PCM) during transportation of newborns with hypoxic ischemic encephalopathy (HIE).

**Study design:**

Randomized controlled trial of newborns with HIE from June 2016 to December 2019. Patients were randomized to transport with PCM or without PCM (control) when transferred to a cooling center in northern Vietnam. Primary outcome measure was mortality rate, secondary outcomes including temperature control and adverse effects.

**Result:**

Fifty-Two patients in PCM-group and 61 in control group. Median rectal temperature upon arrival was 34.5 °C (IQR 33.5–34.8) in PCM-group and 35.1 °C (IQR 34.5–35.9) in control group (*p* = 0.023). Median time from birth to reach target temperature was 5.0 ± 1.4 h and 5.5 ± 1.2 h in the respective groups (*p* = 0.065). 81% of those transported with PCM versus 62% of infants transported without (*p* = 0.049) had reached target temperature within the 6-h timeframe. There was no record of overcooling (< 32 °C) in any of the groups. The was no difference in mortality rate between the two groups (33% and 34% respectively (*p* > 0.05)).

**Conclusion:**

Phase-change-material can be used as a safe and effective cooling method during transportation of newborns with HIE in low-resource settings.

**Trial registration:**

The study was retro-prospectively registered in Clinical Trials (04/05/2022, NCT05361473).

## Background

Neonatal hypoxic-ischemic encephalopathy (HIE), caused by a lack of blood flow and oxygen to the brain at birth, occurs in 10–20/1000 live births in low-middle-income countries (LMICs) [1]. In Vietnam, birth asphyxia accounts for 14% of all neonatal mortality [2], with regional variation and reported rates as high as 33% in certain regions [3, 4]. Although the incidence of birth asphyxia in Vietnam is high, there is still limited experience with therapeutic hypothermia (TH) treatment, as in other LMICs.

Meta-analysis of studies from high-income countries (HICs) show that induced hypothermia is associated with reduced risk of death or major neurodevelopmental disability by 18 months of age [5, 6]. The benefits of cooling, however, is insufficiently studied and requires further evaluation in LMICs [7, 8].

Evidence from animal and newborn studies has indicated that the window of opportunity to start cooling is within 6 h after birth [9, 10]; hence, transportation time could delay hypothermia treatment, consequently altering the safety or effectiveness of TH treatment and neurological outcomes [11].

In order to optimize the neuroprotective effect in newborns with HIE, cooling should start as early as possible, both actively and passively. Several studies have shown that passive cooling (e.g., removal of clothes, turning off heating devices to allow the baby to cool down naturally) is less effective and has a higher risk of overcooling compared to active cooling using servo-controlled equipment [12–14]. Despite the demonstrated benefits of servo-controlled devices when used in transport [13, 15] more recent studies continue to show achieving target temperature within the 6-h window remains a challenge for THs even in HIC settings with proportions between 55 and 63% [16] let alone in LMICs like Vietnam.

Phase change material (PCM) has been well studied in both animals and newborn infants for its effectiveness and safety in therapeutic hypothermia treatment [17, 18]. A PCM-based device has been shown to be comparable to standard servo-controlled equipment in maintaining the target temperature [19, 20]. A pilot study implementing PCM to cool asphyxiated newborns in Vietnam has proven that it is an effective yet easy to use method of cooling in a low-resource neonatal intensive care unit [21].

Following our previous study, the aim of this study was to determine the feasibility and effectiveness of phase-change-material mattresses during transportation of asphyxiated newborns with suspected HIE, with the aim of reducing the time to initiate therapeutic cooling in low-resource settings.

## Participants and methods

### Participants

Following a pilot study showing that PCM is feasible and safe for inducing TH [21], we conducted a randomized controlled trial study of patients from seven hospitals with up to 200 km transport distance to a tertiary center. The inclusion criteria were infants ≥ 36 weeks gestational age and ≤ 6 h after birth with either Apgar score ≤ 5 at 10 min or continued need for resuscitation at 10 min or pH < 7.0 and/or base deficit > 16 mmol/L and sign of moderate to severe encephalopathy (altered consciousness, abnormal tone). Infants were excluded if they were > than 6 h old at the time of referral/evaluation, had coagulopathy with active bleeding, were prenatally diagnosed with syndromes, or had malformations or metabolic disorders not compatible with survival [22]. Prior to the study, visits to assess the hospital and training sessions had been organized to enhance neonatal unit doctors and nurses’ knowledge at all sites concerning the background of the study, the use of a PCM mattress, data recording, and assessment of patients. Parents/guardians of asphyxiated newborns were approached regarding participation in the study once the patient met criteria for cooling. This study was intended to be a feasibility study to assess the safety and applicability of using PCM for transportation of asphyxiated newborns compared to passive cooling (standard care). According to our pilot study, the mortality rate of HIE infants was 35% [21], sample size was calculated showing that 67 infants in each group would be sufficient to give a 95% confidence level with a power of 80% to detect a difference of 15% mortality rate between the two groups. We therefore planned to recruit 20 patients in each of the seven study sites, giving a total of 70 infants per group.

### Study design and intervention

After delivered at provincial hospital, the babies were stabilized by local team and if cooling criteria were met, local team will contact the tertiary hospital in request for transfer.

**Fig. 1:**
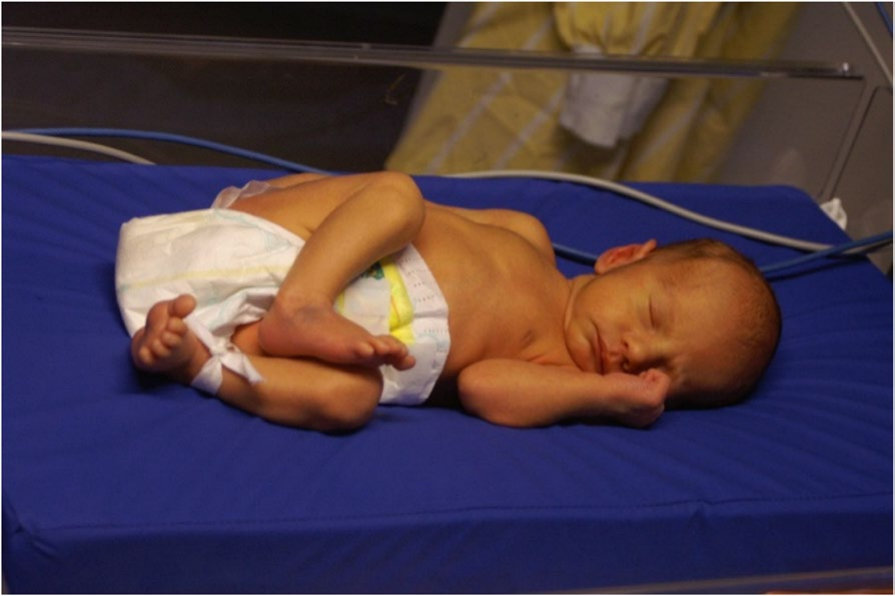
Illustration of an infant on PCM mattress

Each hospital was provided with sealed envelopes containing information for PCM or control by the study team. Randomization was stratified by each of the 7 referring hospitals to one of the two groups. One group cooled by PCM mattress *(Medical Cooling Sweden AB; by TST AB, Kinna, Sweden)* during transportation. The PCM mattress that was used contained 8 PCM packs with a specific melting point of 32°C, built into two layers of 4 × 2 PCM sheets. The mattress was covered with a fabric with possibilities for providing temperature changes between the PCM and the patient through thermal conductance. The newborn is positioned on the PCM mattress, ensuring optimal contact with the cooling surface and secure fixation during transport (Illustrated Fig. 1).

In the control group, patients were passively cooled by turning off all external heating sources. The babies were transported in road ambulances, placed on an ambulance stretcher with only one layer of clothes on, no extra cover by blanket (referring hospitals do not have transport incubators) during transportation, which enabled the babies to chill spontaneously at air temperature.

Axillary temperature and environment temperature were measured before transport and every 30 min during the transportation for both groups. The infant’s condition was clinically monitored by the accompanying hospital staff, but there was no continuous monitoring of heart rate or oxygen saturation during transport. On arrival at VNCH, the infants were evaluated for continuation of cooling or initiation of cooling, respectively. All received hypothermia treatment with PCM mattress.

Both groups received cooling therapy with a target temperature of 33.5–34.5 °C for 72 h and standard medical care. On admission, the severity of the infant’s encephalopathy was assessed by modified Sarnat staging while Thompson score was used daily to assess the clinical progress of the newborn. It involves evaluating various clinical signs such as level of consciousness, tone, reflexes, seizures, and respiratory function, with each sign scored to provide an overall severity rating [23]. Short-term adverse effects of cooling, such as respiratory cardiovascular complications (bradycardia, hypotension, pulmonary hypertension), electrolyte imbalance (hypokalemia, hyponatremia), and coagulopathy – platelet dysfunction, were recorded during treatment.

### Outcome measures

In this study, the primary aim was to evaluate the effectiveness of the phase-change-maternal (PCM) mattress in the transportation of newborns with hypoxic-ischemic encephalopathy (HIE). The primary outcome measure was mortality, serving as a critical indicator of the PCM mattress’s overall impact on patient survival. Secondary outcomes included rectal temperature on arrival, time to reach target temperature 33.5 °C (postnatal age), neurology assessment on arrival and at discharge (for survivors), Short term adverse effects of cooling included: bradycardia (heart rate < 100 beats per minute, hypotension (mean arterial blood pressure < 10th percentile for gestational age), persistent pulmonary hypertension (diagnosed clinically and with echocardiography), hypokalemia (serum potassium level < 3.5 mEq/L), hyponatremia (serum sodium level < 135 mEq/L) and coagulopathy (bleeding and depletion of anti-coagulant factors). By examining mortality, temperature control, and other relevant outcomes, we aimed to provide a holistic evaluation of the PCM mattress’s effectiveness in improving the care of newborns with HIE during transport.

### Statistical analyses

Demographic factors and clinical characteristics were summarized with counts (percentages) for categorical variables, mean (standard deviation [SD]) for normally distributed continuous variables or median (interquartile or entire range) for other continuous variables. For each group, differences were assessed using Student’s t test (normal distribution) or the Mann Whitney U test (skewed distribution). Data were analyzed by using SPSS. *P* value < 0.05 was considered statistically significant.

## Results

**Fig. 2:**
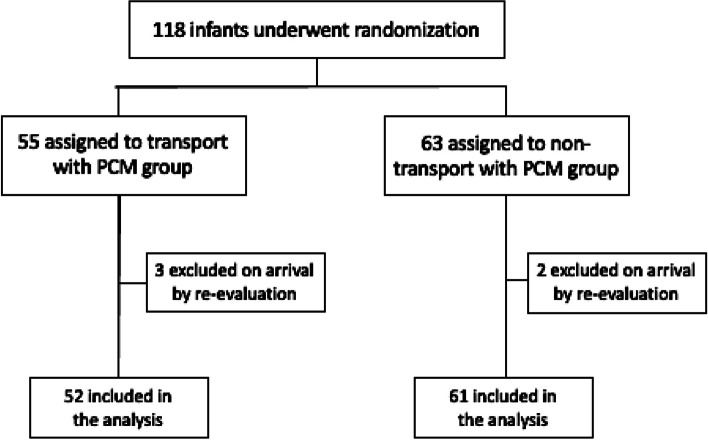
Enrolment, Randomization of the study participants

During the three-year study period (from September 1st 2016 to December 31st 2019), there were in total 118 infants transferred from the seven study hospitals: 55 in the PCM group, and 63 in the control group. The average time for transfer in PCM group and control was 2.1 h (0.5–2.8) and 2.2 h (0.5–3) respectively. Upon arrival at VNCH, during re-evaluation, the encephalopathy was mild in five patients (3 in PCM group, 2 in non-PCM group), and cooling was therefore stopped for these children, and they were excluded from the analysis. The study participants are summarized in Fig. 2.

Baseline maternal and neonatal characteristics for both groups are presented in Table 1.

**Table 1:** Maternal and neonatal baseline characteristics. Figures are numbers (percentages) and means (standard deviation)

Characteristic	PCM group (*n* = 52)	Control group (*n* = 61)	*P* value
**Maternal**			
Age – years	27 ± 5	26 ± 6	0.160
Intrapartum complications – no. (%)	43	44	0.083
Fetal heart-rate deceleration	15 (29)	18 (34)	
Cord prolapses	2 (4)	0 (0)	
Shoulder dystocia	3 (6)	2 (4)	
Maternal hemorrhage	2 (4)	3 (6)	
Mode of delivery – no. (%)			0.671
Vaginal delivery/Instrumental Delivery	28 (54)	34 (56)	
Emergency caesarean delivery	24 (46)	27 (44)	
**Neonatal**			
Male – no. (%)	37 (71%)	39 (64%)	0.522
Gestational (weeks)	39 (± 1.1)	39 (± 1.2)	0.634
Birthweight (grams)	3210 (± 1040)	3165 (± 416)	0.754
Apgar score ≤ 5 – no. (%) at 10 min^a^	22 (100%)	28 (100%)	
Age at admission (hours from birth)	3.6 ± 1.4	3.9 ± 1.4	0.119
Transport distance, hours by road ambulance (Median, range)	2.1 (0.5 – 2.8)	2.2 (0.5 – 3)	0.204
Time to reach target temp 33.5 °C to 34.5 °C – (hours from birth)	5.0 ± 1.4	5.5 ± 1.2	0.065
Number of patients reaching target temp within 6 h of birth – no. (%)	42 (80.8%)	38 (62.3%)	0.049
Rectal temp at randomization, before transport (°C)	35.8 ± 0.6	36 ± 0.8	0.125
Seizure on admission – no. (%)	5 (10)	3 (5)	
Level of encephalopathy – no. (%)			0.625
Moderate—Sarnat stage II (n)	18 (35)	23 (38)	
Severe—Sarnat stage III (n)	34 (65)	38 (62)	

^a^Apgar score at 5 min and 10 min were available for only 22 newborns in PCM group and 28 newborns in control group

Newborns had a mean gestation of 39 (± 1.1) weeks in both groups, and the majority were male, with 37 (71%) in the PCM group and 39 (64%) in the control group. There was no significant difference between the two groups in terms of maternal age or mode of delivery. Close to 50% of cases required emergency c-section due to intrapartum complications such as fetal distress, cord prolapse, bleeding, or other complications. Eligibility criteria for transportation and evaluation for cooling were adapted to previous multicenter studies [22] and based on either Apgar scores or need for continuation of resuscitation at 10 min, because blood gases were not routinely reported or assessed.

In the PCM group, 15 (29%) newborns had clinical seizures on admission, with a mean age at admission of 3.6 h ± 1.4. In the non-PCM group, 23 (38%) newborns had clinical seizures on admission, with a mean age at admission of 3.9 h ± 1.4. Anticonvulsant use was similar in both groups.

**Fig. 3:**
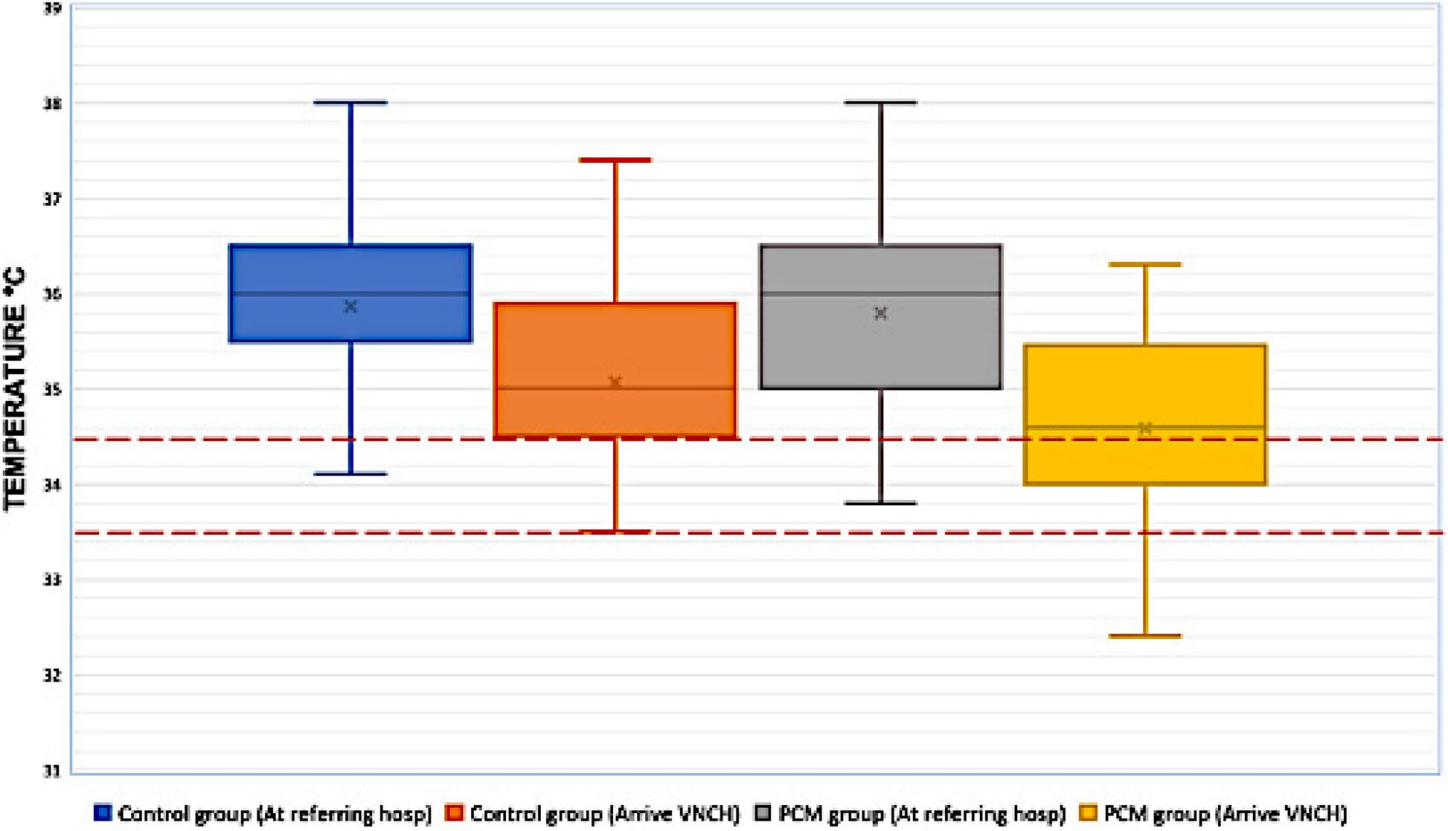
Box plot for temperature measurement at referring hospital and on arrival at VNCH. The area between the redlines shows the target temperature range of 33.5 °C to 34.5 °C

Figure 3 shows the mean temperature of the newborns in both groups at two different time points: on departure from the referring hospital and on arrival at VNCH. Upon arrival to VNCH, the mean rectal temperature in the PCM group was significantly lower than in the control one: 34.5 °C (IQR 33.5 – 34.8) compared to 35.1 °C (IQR 34.5 – 35.9) (*p* = 0.023). There was no record of excessive hypothermia (< 32 °C) on admission for any of the groups. Consequently, the median time to reach target cooling temperature from birth in the PCM group was 5 ± 1.4 h, while it took 5.5 ± 1.2 h for the control group to reach the target cooling temperature (*p* = 0.065). 42/52 patients (81%) of those transported with PCM versus 38/61 (62%) of infants transported without (*p* = 0.049) had reached target temperature within the 6-h timeframe.

There was no significant difference in Thompson scores between the two groups on arrival and on consequent days.

In total, 38 newborns died before discharge from hospital: 17 in the PCM group and 21 in the control group. In the PCM group, the newborns died from the following causes: encephalopathy-related complications (*n* = 13 [74%]), persistent pulmonary hypertension (*n* = 3 [18%]), and sepsis (*n* = 1 [6%]). In the control group, the primary cause of death was also encephalopathy-related complications (*n* = 18 [86%]); other causes including persistent pulmonary hypertension (*n* = 2 [4%]) and pneumothorax (*n* = 1 [2%]). The proportion of deaths according to Sarnat stage was similar across groups (Table 2). Among the 75 survivors, 69 (92%) completed follow-up until 18 months. Nineteen children developed cerebral palsy (8 diplegia, 3 hemiplegia, 8 dyskinetic), and 11 had delayed neurodevelopment [24].

**Table 2:** Clinical outcomes

Variables	PCM group (*n* = 52)	Control group (*n* = 61)	*P* value
Encephalopathy score (Thompson score)			
Day 1, Mean, min–max	16 (9 – 21)	17 (9 – 22)	n.s
At discharge, Mean, min–max	5 (0 – 18)	6 (0 – 18)	n.s
Complications during cooling – no. (%)			
Hypotension ± inotropes	30 (58)	33 (54)	n.s
Pneumothorax	2 (4)	0	n.s
Uncontrollable seizures	4 (8)	3 (5)	n.s
Sepsis	10 (19)	10 (16)	n.s
MRI at day 7–10 – no. (%)			
White matter injury	10 (19)	11 (18)	n.s
Cerebral hemorrhage	3 (6)	1 (2)	n.s
Brain edema	2 (4)	1 (2)	n.s
Brain atrophy	0	1 (2)	n.s
Deaths – *n* (%)			
Total	17 (33%)	21 (34%)	n.s
Sarnat stage II	2	3	n.s
Sarnat stage III	15	18	n.s

## Discussion

In this randomized controlled study, we show that phase changing material (PCM) can be used as a safe and effective cooling method during transportation of newborns with HIE. The mortality rate was similar between the two groups; however, the study was underpowered to detect a difference between two groups. Target temperature was reached faster in the group where PCM was used during transportation, and there were no complications during transportation.

In the present study, the overall mortality rate was 34%, which was significantly higher in comparison to 19% in previous trials from high-income countries [25]. However, the mortality rate was similar to other LMIC settings, such as Nepal (31%) [26], and even slightly lower than, e.g., India, Sri Lanka, and Bangladesh (42%) [8]. The proportion of deaths according to Sarnat stage was similar across groups, and up to 50% of infants with Sarnat stage III encephalopathy died. Indeed, studies from Nepal have shown that, in settings where long-term ventilation and stabilization are not available, the mortality rate for newborns with Sarnat stage III neonatal encephalopathy can be as high as 100% [26]. Comparable to many cooling studies, there were no differences between the two groups in the present study in terms of adverse events, such as hypotension, arrhythmias, coagulation dysfunction, skin injury due to cooling, or infection [27].

The results from our study, with combined infant mortality and severe neurological disability of 55% in infants treated with TH [24], were comparable to both the results in the randomized HELIX trial, in part performed during the same time. Possible explanations for the worse outcomes in LMICs include the larger proportion of newborns requiring transport and referral from other hospitals, longer duration of the hypoxic insult, and a higher proportion of newborns having clinical seizures, all of which are comparable to the findings of this study. Since there are multiple factors that could influence the results of hypothermic neuroprotection, the question of whether we should stop cooling newborns with HIE in LMICs should be investigated further. There are many differences in the epidemiology and outcome of newborns with encephalopathy in low-resource settings such as higher incidence of hospital-acquired infections like sepsis or pneumonia in Vietnam’s hospitals [28]. Additionally, there is convincing evidence that suggests that a combination of infection and ischemia results in more severe brain injuries and increases in the risk of adverse outcomes [29, 30], which is possibly one of the factors responsible for the poorer neurological outcome reported from low and mid-resource settings [26]. Among the 113 patients in this study, we suspected early-onset sepsis by history in 10 patients, only 2 patients (0.2%) were diagnosed with sepsis by positive blood cultures. In our study, none of the children had documented metabolic disorders.

The transfer of patients took 2.1 h (0.5 – 2.8) in PCM group and 2.2 h (0.5 – 3) in control, with a median temperature on arrival 0.6 °C lower in the PCM group (34.5 °C) than in the control group (35.1 °C). In a subtropical region like Vietnam, average temperatures range from high of 35.6 °C (96.1°F) to low of 14.6 °C (58.3°F), with an average yearly temperature of 24.8 °C (76.7°F)”. Under such thermal conditions, passive cooling may be less effective than more efficient methods of cooling. When comparing the temperature on arrival with other studies from similar settings, it was lower than in both the NICHD trial (36.6 ± 1.0 °C) [25], where all patients were inborn, and in the Thayil S et al. trial (35.2 ± 1.3 °C) [20]. The target temperature of 33.5 °C was achieved at a median of 1.5 h earlier for the PCM group than the control group, which is a possible advantage, since studies have shown that reaching the target temperature early is often associated with better motor outcomes at 18 months in surviving newborns [10]. In a large cohort of 207 infants in Canada who received cooling for HIE, it was shown that initiating cooling before and during transfer is critical for achieving the target temperature sooner, which is a key factor for improving outcomes [31, 32]. Lemyre et al. also suggested that the severity of the encephalopathy was associated with the time to reach the target core temperature. We did not demonstrate a statistically significant difference in time to reach target temperature between groups (*p* = 0.062). However, we did demonstrate a significant difference in the proportion of infants reaching target temperature by 6 h of age between groups (80.8% in infants transported with PCM versus 62.3% of infants transported without, *p* = 0.049). This difference means that a larger proportion of infants received neuroprotection within the narrow six-hour timeframe shown to be efficacious in animal and human studies of TH for HIE. Transport over both short and long distances makes achieving and maintaining target temperature a great challenge globally. Studies in both LMICs and HICs show that delays in the initiation of cooling therapy (even though still within the six-hour window of the protocol) result in longer time to attain target temperatures and likely contribute to worse outcomes. [33, 34].

In our study, no patient in either of the two groups developed severe hypothermia (< 32 °C). However, several authors have raised concerns that initiation of cooling during transport may increase the risk of excessive cooling [35, 36] especially when ambulances in most LMIC settings are used solely for transport and not as an emergency care vehicle, due to poorly equipped facilities and lack of trained medical staff [37, 38]. In comparison, removal of an external heat supply in 18 transported newborns in Sweden resulted in 3 (17%) newborns becoming overcooled to below 32 °C [36]. Similarly, Zanelli et al. reported that in their study of 11 newborns, two newborns were significantly overcooled (29.2 °C and 29.6 °C) upon arrival at the hospital by passive cooling. In another study of 35 newborns, where active cooling was conducted by placing cool packs in an incubator, 34% of newborns had core temperatures below 32 °C on arrival at the cooling center [39].

## Conclusion

Initiating therapeutic hypothermia with a PCM mattress during ambulance transportation is feasible and safe, allowing for significantly earlier initiation and attainment of target temperatures. While this approach shows promise, further research is needed to definitively determine its potential benefits for neonates with hypoxic-ischemic encephalopathy, as current evidence primarily comes from animal studies.

## Trial registration

The study was retro-prospectively registered in Clinical Trials (04/05/2022, NCT05361473).

This study was conducted in strict accordance with the Consolidated Standards of Reporting Trials (CONSORT) guidelines. All aspects of the study, including participant enrollment, allocation, intervention, follow-up, and data analysis, were designed, and reported to ensure transparency, rigor, and reproducibility in line with the CONSORT recommendations. Detailed checklists have been provided as supplementary materials to illustrate the study’s adherence to these standards.

## Data Availability

The data and material used in this study are available from the corresponding author on reasonable request.

## Abbreviations

HICs: High-income countries
HIE: Hypoxic ischemic encephalopathy
LMICs: Low-middle income countries
NICU: Neonatal intensive care unit
PCM: Phase change materials
TH: Therapeutic hypothermia
VNCH: Vietnam National Children’s Hospital
